# The safe screw path along inferior border of the arcuate line at acetabular area: an anatomical study based on CT scans

**DOI:** 10.1186/s12891-017-1453-0

**Published:** 2017-02-20

**Authors:** Chun Bi, Jiandong Wang, Xiaoxi Ji, Zhijian Ma, Fang Wang, Xiangsen Zeng, Dongmei Wang, Qiugen Wang

**Affiliations:** 1 Trauma Center, Shanghai General Hospital, School of Medicine, Shanghai Jiaotong University, 650 Xin Songjiang Road, Shanghai, 201620 People’s Republic of China; 2 Department of Orthopedic Surgery, the Second People’s Hospital of Yunnan Province, Yunnan, China; 3 School of Mechanical Engineering, Shanghai Jiaotong University, Shanghai, People’s Republic of China

**Keywords:** Digital anatomical measurement, Acetabular fracture, Safe screw insertion, Inferior border of arcuate line

## Abstract

**Background:**

Misplaced screw during the internal fixation of acetabular fractures may penetrate the hip joint which might cause chondrolysis and traumatic osteoarthritis in the future. This study aims to acquire the safe path for screw insertion along inferior border of the arcuate line fixation route at acetabular area.

**Methods:**

Computed tomography (CT) scans of 98 patients without pelvic trauma were rebuilt for three-dimensional models of pelvis. After depicting the fixation route curve, five cross-sections perpendicularly to the curve were established from the anterior of pelvis to the posterior along inferior border of the arcuate line. The safe screw lengths for section 1 and 5 were measured from the computer models. In section 2, 3 and 4, a line from the screw entry point tangent to the inferior edge of the acetabulum was depicted and the measurements of minimum safe direction of screw insertion were performed then marked with angle θ.

**Results:**

The safe screw lengths for section 1 and 5 were 22.29 ± 4.41 mm and 32.64 ± 4.70 mm (*n* = 98). The minimum safe angles of screw insertion for the middle three sections 2, 3, and 4 were 65.38 ± 10.23°, 74.20 ± 10.20°, and 57.88 ± 11.11°(*n* = 98), respectively. The results for the male group (*n* = 98) indicated smaller minimum safe angles in these three sections compared with the female (*n* = 98).

**Conclusions:**

Compared to male, the minimum safe angles of screw placement at acetabular area for female should be more away from inferior edge of acetabulum and tilt to the bottom of pelvis along inferior border fixation route in surgical management of acetabular fractures.

## Background

Rigid and accurate internal fixation is required for acetabular fractures with displacement to achieve anatomical reduction and good prognosis [1, 2]. However, because of its complicated anatomical structures, surgical management of acetabular fractures still faces challenges. Due to the difficulty of viewing the articular surface during the internal fixation of surgical procedures, there might be a possibility of screw penetration into the acetabulum which frequently results in traumatic osteoarthritis in the future [3], and it would seriously affect the life quality of these patients. Hence, understanding the sophisticated anatomical structures of acetabular area plays a significant role in providing the guidance for orthopedic surgeons to avoid intra-articular screw insertion.

The Stoppa and the illoinguinal approaches, classical anterior approaches, are both commonly employed for treating acetabular fractures [4]. Previous study has provided the reference for the effective screw insertion along the superior border fixation route at acetabular area [5]. This area can be exposed via the illoinguinal approach. However, it just provides limited access to the quadrilateral surface and the posterior column. Meanwhile, the high risk of injures to major blood vessels,particularly the Corona Mortis, via this approach, was the main limitation of this procedure [6, 7]. In contrast, the Stoppa approach allows a widely and directly view of quadrilateral area, anterior column as well as the arcuate line [8]. Through superior border approach, clearly exposure of the anterior wall and anterior column could be obtained while through inferior border approach not only the aforementioned structures and whole quadrilateral surface can be better exposed, but also avoiding the dangerous Corona Mortis to be damaged. Compared with the superior border area, the area of inferior border is more suitable for placing the plate for fixation of acetabular fractures especially with quadrilateral surface and posterior column damaged [9]. Although some previous researches with cadaveric bone provided parameters on safe screw insertion, there inevitable had some level of errors with these artificial measurements [10–12]. Meanwhile, with relative small sample sizes, the whole conditions of individuals cannot be efficiently estimated. Digital three-dimensional (3D) reconstruction and measurement, as a high efficient method for orthopedic anatomic studies, has outstanding advantages in accurate measuring compared with those traditional measurements [13].

Based on 3D reconstructions and measurements of pelvic CT scans, a retrospective observational study was performed. The objective of the present study is to acquire safe path for screw insertion along the inferior border fixation route at acetabular area.

## Methods

### Patients and models reconstruction

Ninety-eight patients including 60 males and 38 females without pelvic trauma or deformity, diagnosed with varicose veins in lower extremity, were recruited in Shanghai General Hospital from December 2009 to November 2010. The mean age of these available samples was 60.1 years (range: 22–91 years, Table 1). Shanghai General Hospital’s Review Board approved this study.

**Table 1:** Age distribution of all 98 patients

	21–30 years	31–40 years	41–50 years	51–60 years	61–70 years	71–80 years	≥81 years
Male	3	10	2	17	12	11	5
Female	1	1	7	11	7	7	4
Total	4	11	9	28	19	18	9

All these samples underwent a 64-channel computed tomography (CT) scan. To obtain clearly pelvic images, the needed thickness and scanning time for each slice was 0.75 mm and 0.2 s, respectively. Stored with DICOM format, all raw imaging data of each patient were then processed by Mimics 13.0 where three-dimensional (3-D) pelvic model was reconstructed. After that, thresholding segmentation, region growing and surface smoothing were performed to the 3-D pelvic model and saved with Stereo Lithography (STL) format. Then, the Geomagics 10.0 software was used for further smoothing and noise reduction.

### Essential parameters’ measurements

In the beginning, via Geomagics 10.0 software, three fundamental planes defined as the sagittal, coronal and horizontal plane were set up, respectively. The sagittal plane was recognized as the plane through from the pubic symphysis to the midpoints of the whole sacrum and the coccygeal tip. In coronal plane, the anterior superior iliac spine and pubic tubercle were positioned at the same level to comply with anatomical position during the process of the study. Perpendicular to both above-mentioned planes, the horizontal plane was established. A curve was delineated by collecting some points, 5 mm lateral and inferior to the pelvic rim, along the inferior border fixation route from pubic tubercle, pubic ramus inner side, arcuate line, to sacroiliac joint. The site with distance of 5 mm at this area is frequently used as the entry point of screw. Saved with STL format, all these objects were imported to the Imageware 23.0 where an optimal ball fitting the acetabular fossa was established. And after being saved with IMW format, the whole model was imported to the Unigraphic NX 7.0.

**Fig. 1:**
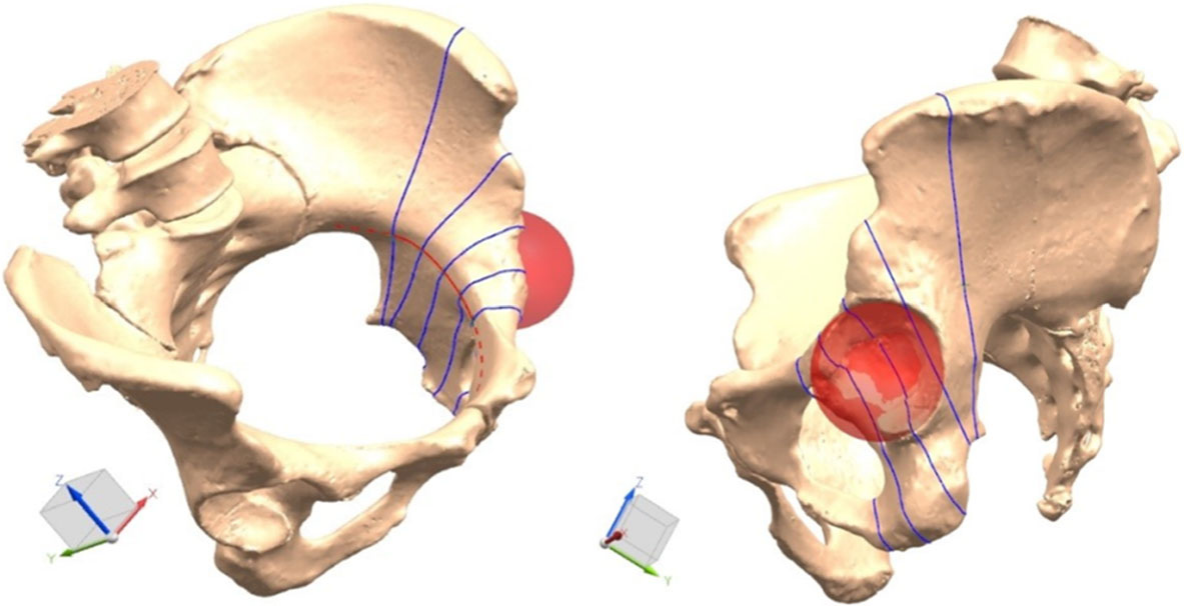
Perpendicular to the space curve, five acetabular area sections from pubic tubercle to sacroiliac joint were acquired

**Fig. 2:**
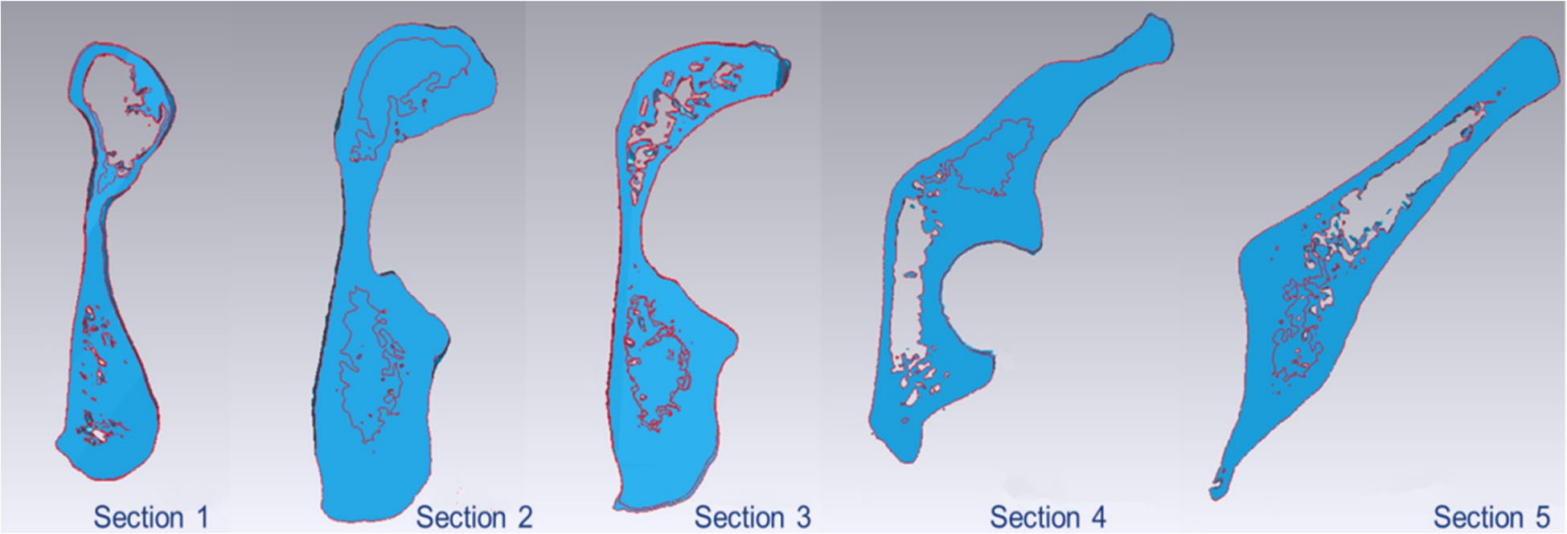
Marked as section 1, 2, 3, 4, and 5, these five sections were listed respectively

Passing through the center of the ball, a section perpendicular to the space curve was built as the reference plane. Parallel to this section, two planes with interval of the quarter of diameter forwardly and backwardly along the space curve were built, respectively. These five planes segmented the space curve at acetabular area into four equal parts. Thus, five intersection points between each plane and the curve were acquired as the screw entry points (Fig. 1). Through these five intersection points, five acetabular area sections perpendicular to the space curve from pubic tubercle to sacroiliac joint were acquired then marked as section 1, 2, 3, 4, and 5, respectively (Fig. 2).

**Fig. 3:**
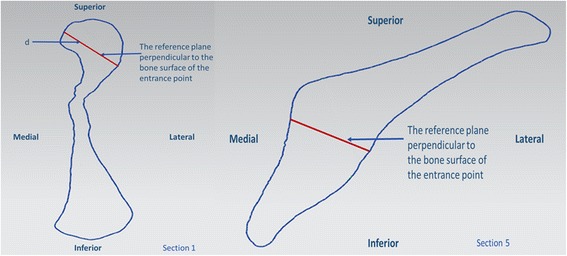
For section 1 and 5, screws can be placed perpendicularly to the bone surface directly. The measured distance between the screw entry point and the intersection point was recorded as the screw length, d

**Fig. 4:**
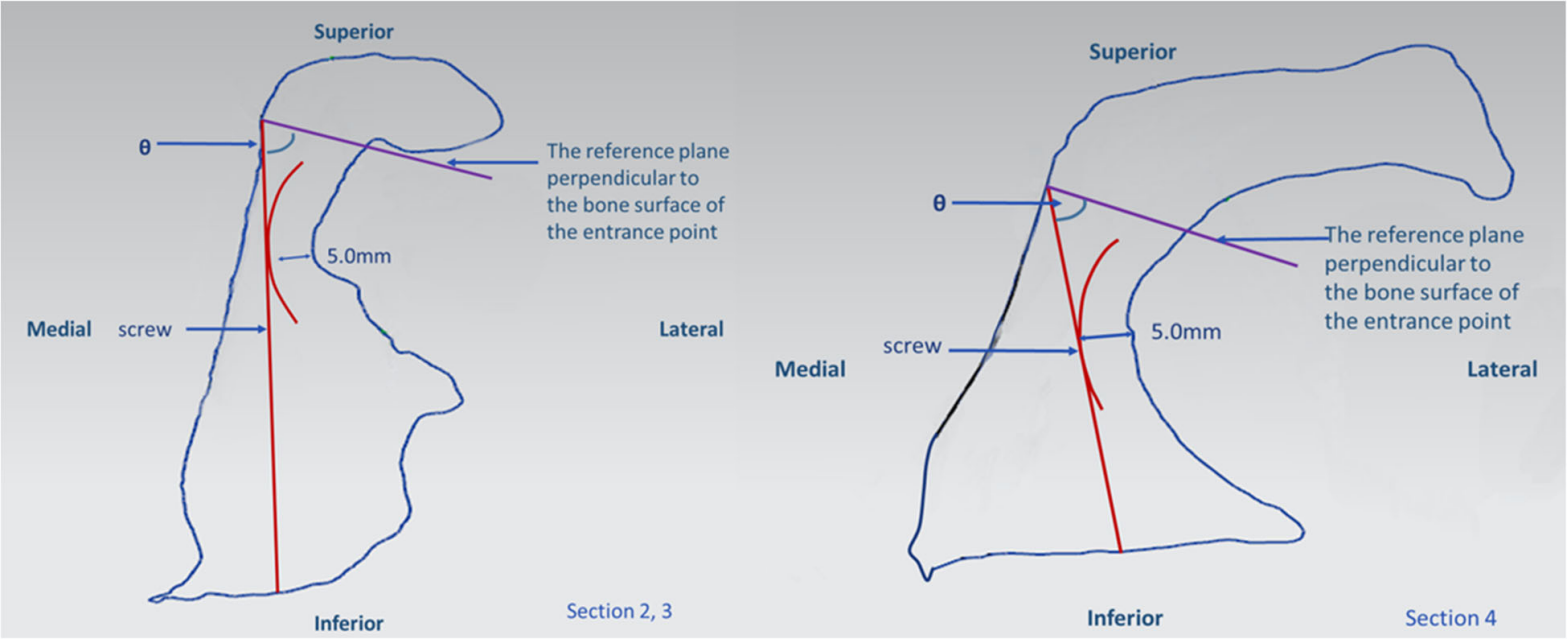
For section 2, 3 and 4, a line through the screw entry point and tangent to the lower margin of the arc was defined as the screw-placement direction. The angle between this tangent line and the normal plane was marked as θ. Due to the similar shaping, the measurement for section 2 and section 3 was represented with the same model

Through the screw entry point of each section and perpendicular to the cortical surface, another reference plane was built. The section and reference plane intersected at one point which was recorded as the intersection point. In section 1 and 5, located in the outermost of acetabular area, screws can be placed perpendicularly to the bone surface directly and there is no need to worry about screw penetration into the hip joint. In section 2, 3, and 4 with the shape similarity, the same measurement methods were performed. With several points selected along the edge of acetabulum then expended by 5.0 mm, an arc was delineated to represent the subchondral bone thickness. A line through the screw entry point and tangent to the inferior edge of the arc was defined as the screw-placement direction. Formed by the tangent line and the normal plane, the angle was marked as θ. The distance, defined as the screw length d from the screw entry point to the intersection point, was measured in section 1 and 5. Due to the similar shaping, the measurements for section 2 and section 3 were represented with the same model (Figs. 3 and 4).

### Statistical analysis

Data were reported as mean ± standard deviation. Normality tests were used for analysis of data distribution and *t*-test was used for analyzing the mean values of different groups. Significance was set at a level of *p* < 0.05. All statistical analyses were performed using the SPSS software (version 19.0).

## Results

The experimental results showed the differences on minimum safe angles and safe screw lengths between the male and female in different sections. In section 2, 3, and 4, the minimum safe angles were 67.40 ± 7.11° (*n* = 98), 72.66 ± 9.35° (*n* = 98), and 57.88 ± 11.11° (*n* = 98), respectively. The results for the male group (*n* = 98) indicated smaller minimum safe angles in these three sections compared with the female (*n* = 98). Significance of differences between male and female groups for θ in section 2, 3 and 4 were indicated (*P* = 0.012, *P* = 0.032 and *P* = 0.025, respectively). The safe screw lengths for section 1 and 5 were 21.94 ± 3.07 mm (*n* = 98) and 33.01 ± 4.47 mm (*n* = 98). The differences of screw lengths between male and female groups in section 1 and 5 were not statistically significant (*P* = 0.08 and *P* = 0.076, respectively) (Table 2).

**Table 2:** Comparison of gender differences

	Section 1	Section 2	Section 3	Section 4	Section 5
	d (mm)	θ (°)	θ (°)	θ (°)	d (mm)
Total	21.94 ± 3.07	67.40 ± 7.11	72.66 ± 9.35	57.88 ± 11.11	33.01 ± 4.47
Male	22.38 ± 2.97	65.95 ± 6.50	71.08 ± 9.74	55.92 ± 10.23	33.64 ± 4.92
Female	21.27 ± 3.15	69.75 ± 7.51	75.25 ± 8.17	61.07 ± 11.87	31.99 ± 3.43
*P* value	0.08	0.012	0.032	0.025	0.076

## Discussion

The special anatomical morphology of acetabular area is more complex and irregular compared with the long bone which increases the difficulties for surgical treatment. Although considerable achievements have been obtained for management of acetabular fractures in recent years, such fractures remain challenging to orthopedic surgeons [14]. During the surgery of acetabular fractures, impossible viewing the joint surface is frequently at risk for screw penetration, which will ultimately bring chondrolysis and osteoarthritis and lead to the complete destruction of the hip joint cartilage [15, 16]. Realizing these, surgeons have debated for a long time to determine the safe methods for screw insertion at this area in order to prevent the occurrence of these complications at the greatest extent.

As two main approaches, the Stoppa approach and the ilioinguinal approach are widely employed in the fixation of acetabular fractures [4]. Our previous study provided the parameters for safe screw insertion of acetabular fractures along the superior border fixation route, and this area can be exposed via ilioinguinal approach [5]. Compared with ilioinguinal approach, Stoppa approach has an evident superiority of allowing widely view of the anterior column, the arcuate line and the quadrilateral surface [8]. With Stoppa approach, it can also avoid the dangerous Corona Mortis to be damaged [6, 7]. Our present study concentrates on safe screw insertion along the inferior border fixation route at the area of acetabulum. Meanwhile, this area is an ideal place for screw insertion when treating acetabular fracture especially with the damaged quadrilateral surface and posterior column.

The safe screw insertion angles can be found from the comparisons between male and female. The male group showed smaller minimum safe angles obviously in the middle three sections. Compared with male, angles for the female should be more away from inferior edge of acetabulum. Thus, it can be interpreted that during the operation, more tilt to the bottom of pelvis should be required for the female to avoid intra-articular screw insertion. And proper length screws used in middle three sections should be selected in accordance with the actual depth detecting during the surgery procedure. Unlike other human bones, the pelvic shape appears markedly different between the male and female. For the general pelvis, there are four different shapes termed gynecoid, android, platypelloid and anthropoid. That means although these samples were divided into two main groups, male and female, because of the overlap of pelvic shapes in these two groups, the results inevitable appear overlapped to some extent. The finding concluded by us provides a general guidance for the surgeons. However, before the surgery, a 3D geometrical reconstruction should also be performed to the patient so as to acquire the accuracy. Thus, a combination of the results of the current study and the preoperative 3-D geometrical reconstruction would provide a relatively accurate guidance in the planning of the surgery.

Focusing on safe path of screw insertion, previous researchers have reported several conclusions with different methods. Xianquan Wang [17] sectioned hemi-pelvises of cadaveric specimen and formed different sections from anterior to the posterior. The maximum medial angulation to provide safe cortical screw insertion at 0.5-, 1.0-, and 1.5-cm entry points is 8.2 ± 2.2°, 14.9 ± 3.4°, and 26.1 ± 4.5°, respectively.^.^ However, it didn’t set up an operative reference plane, thus the relative position between surgeon and entry point might be changed. In our study, perpendicular to the entry point of the cortical surface, the reference plane was built in each section which could be convenient for the surgeons to get the accurate screw insertion during the operation. Ebraheim et al. [11] performed a cadaveric anatomical study and demonstrated the danger area at posterior of acetabulum. From perpendicular to the posterior wall to the posterior acetabular margin at 1 cm interval, the medial angulation for safe screw insertion was described. Although providing quantification of the amount of angulation required for safely screws placement at the posterior of the acetabulum, it did not give the accurate parameters for safely screw placement at anterior of the acetabulum which is essential for treating the complex acetabular fractures as well. Using data from patients’ CT images, Guy et al. [18] reported the safety zone for the internal fixation using screw at the quadrilateral surface with Stoppa approaches. Although testing a number of cadavers, it is quite difficult to perform accurate measurements of the pelvis in a standardized body position only using a 2-D methodology. Based on the 3-D reconstruction models, with male and female of wide age ranges, our current study had relatively large samples which would be more representative compared with these cadaveric studies. Additionally, the current study set more practical reference plane for screw insertion that would be easier for surgeons to operate and less influenced by the body position during the procedure of the operation. Via making 3-D reconstruction model precisely, dividing the cross-sections rationally and measuring the parameters for easy-to-operate safe path accurately, the data provided by this study could be used as a theoretical basis for screw insertion of acetabular fractures. Surgeons can take these safe insertion angles and the screw length as reference which could be beneficial for them to select the proper length of screws and make proper direction during the screw insertion process. Before surgery, the surgeons should first consider the 3-D reconstruction image of the patient and then make preparation according to our conclusion. If there have some minor differences, it needs to be mini-adjusted during the surgery since the general information has been provided. Limitation needs to be acknowledged in this study. All data were collected and analyzed from the out-patients in authors’ hospital. Although our study used a relatively large samples which could make the results of measurements accurate, it only can reflect the general situation of the local residents. And if more samples can be collected and analyzed from different regions and hospitals, it would be beneficial to analyze the regional differences of the measurements and make the results much more accurately. Thus, a multi-center investigation should be performed in the future to make results more meaningful.

## Conclusions

To conclude, results of the present study provided the safe angles for screw insertion along the inferior border fixation route at acetabular area. The minimum safe angles for female should be more away from inferior edge of acetabulum and tilt to the bottom of pelvis along inferior border fixation route in surgical management of acetabular fractures. The definition of safe angles for screw insertion with Stoppa approach made it more practical to guide the surgery. On the basis of accurate 3D reconstructions and digital measurements, a reliable reference on safe path of screw insertion for acetabular surgery was offered by this study.

## Abbreviations

3D: Three-dimensional
CT: Computed tomography
DICOM: Digital Imaging and Communications in Medicine
IMW format: Imageware format
STL: Stereo Lithography
